# Photoacoustic Imaging of Breast Microcalcifications: A Preliminary Study with 8-Gauge Core-Biopsied Breast Specimens

**DOI:** 10.1371/journal.pone.0105878

**Published:** 2014-08-25

**Authors:** Ga Ram Kim, Jeeun Kang, Jin Young Kwak, Jin Ho Chang, Seung Il Kim, Ji Hyun Youk, Hee Jung Moon, Min Jung Kim, Eun-Kyung Kim

**Affiliations:** 1 Department of Radiology and Research Institute of Radiological Science, Severance Hospital, Yonsei University College of Medicine, Seoul, Republic of Korea; 2 Department of Surgery, Severance Hospital, Yonsei University College of Medicine, Seoul, Republic of Korea; 3 Sogang Institute of Advanced Technology, Sogang University, Seoul, Republic of Korea; 4 Interdisciplinary Program of Integrated Biotechnology, Seoul, Republic of Korea; 5 Department of Radiology, Gangnam Severance Hospital, Yonsei University College of Medicine, Seoul, Republic of Korea; Northwestern University Feinberg School of Medicine, United States of America

## Abstract

**Background:**

We presented the photoacoustic imaging (PAI) tool and to evaluate whether microcalcifications in breast tissue can be detected on photoacoustic (PA) images.

**Methods:**

We collected 21 cores containing microcalcifications (n = 11, microcalcification group) and none (n = 10, control group) in stereotactic or ultrasound (US) guided 8-gauge vacuum-assisted biopsies. Photoacoustic (PA) images were acquired through ex vivo experiments by transmitting laser pulses with two different wavelengths (700 nm and 800 nm). The presence of microcalcifications in PA images were blindly assessed by two radiologists and compared with specimen mammography. A ratio of the signal amplitude occurring at 700 nm to that occurring at 800 nm was calculated for each PA focus and was called the PAI ratio.

**Results:**

Based on the change of PA signal amplitude between 700 nm and 800 nm, 10 out of 11 specimens containing microcalcifications and 8 out of 10 specimens without calcifications were correctly identified on blind review; the sensitivity, specificity, accuracy, positive predictive and negative predictive values of our blind review were 90.91%, 80.0%, 85.71%, 83.33% and 88.89%. The PAI ratio in the microcalcification group was significantly higher than that in the control group (the median PAI ratio, 2.46 versus 1.11, respectively, P = .001). On subgroup analysis in the microcalcification group, neither malignant diagnosis nor the number or size of calcification-foci was proven to contribute to PAI ratios.

**Conclusion:**

Breast microcalcifications generated distinguishable PA signals unlike breast tissue without calcifications. So, PAI, a non-ionizing and non-invasive hybrid imaging technique, can be an alternative in overcoming the limitations of conventional US imaging.

## Introduction

Mammographic lesions manifested by microcalcifications constitute approximately half of clinically occult breast cancers and are frequently employed as an indicator of early breast cancer [1]–[3]. If microcalcifications detected on mammography are deemed suspicious for malignancy, a biopsy is required and recently, among a variety of biopsy approaches, vacuum-assisted biopsy with stereotactic guidance is mainly chosen. Even though this method is successful in aiding the diagnosis of breast microcalcifications, stereotactic guidance requires mammographic compression and radiation exposure of the breast [4], [5]. In general, ultrasound (US)-guided vacuum-assisted biopsy is preferred over mammographic guidance because US guidance offers a number of advantages over stereotactic guidance: patients can be in a supine position without compression of the breast and radiation hazards; images of the procedures can be acquired in real time [4]–[7]. Although the low visibility of microcalcifications can be one of the biggest drawbacks of US imaging due to its relative low contrast resolution, US-guided vacuum-assisted biopsy can be an effective alternative to stereotactic-guided vacuum-assisted biopsy in the cases where microcalcifications are visible on US [6], [8]–[10].

Since photoacoustic imaging (PAI) is a real-time molecular imaging modality with high spatial and contrast resolutions, it may be an alternative for the imaging of breast microcalcifications in real time [11]–[13]. The PAI technology has been applied to various areas, such as tumor angiogenesis studies, functional brain studies, and deep internal organ imaging in animals [13]–[17]. Previous reports about PAI for real-time detection of breast microcalcifications were performed in order to determine optimal laser wavelength that was found to be 700 nm. In fact, the laser energy absorbance consistently decreases from 700 nm to the near-infrared spectrum range. By using the unique absorbance spectrum, 3-dimensional spectroscopic photoacoustic (PA) images of breast specimens were proposed [18], [19].

In this paper, we present the PAI tool and evaluate whether microcalcifications can be detected on PA images using breast specimens confirmed as benignity or malignancy.

## Materials and Methods

### Study Sample

The study was approved by the Institutional Review Board of Severance Hospital, Yonsei University Health System (4-2011-0823). All patients were informed about their inclusion in the study. Written consent was obtained from all subjects prior to their biopsy procedures.

Among patients who were scheduled to undergo sequential 8-gauge vacuum-assisted core needle biopsy procedures with stereotactic guidance or US guidance for breast microcalcifications suspected to be malignancy by mammography (Breast Imaging Reporting and Data System [BI-RADS] category 4 or 5), 11 patients agreed to participate in our study from April 2012 to March 2013 and they were prospectively included in the microcalcification group (all female; age, 31–74 years). Eleven cores from the 11 patients were obtained and specimen mammography was performed to confirm the presence of microcalcifications in core samples. The control group consisted of 5 patients (all female; age, 24–41 years) undergoing sequential 8-gauge vacuum-assisted core needle excision procedures with US guidance for biopsy-proven fibroadenoma during the same period. These patients were also prospectively included in our control group with their consent. Specimen mammography of the 10 cores obtained from the 5 patients was performed to confirm that the cores of the control group revealed no microcalcifications. In all, 21 cores of breast tissue were obtained from the 16 patients who were included in our study. Mammography was performed using the Selenia full field digital mammography system (Lorad/Hologic, Danbury, Conn). To allow correlation between the two modalities, specimen mammographic images were acquired in 2 view image planes: top-down and lateral view. The number and size of calcifications on each specimen mammography were evaluated. The size of each calcification in the cores of the microcalcification group was assessed using the longest diameter of each calcification focus shown on specimen mammography.

### Imaging System and Experimental Procedures

**Figure 1:**
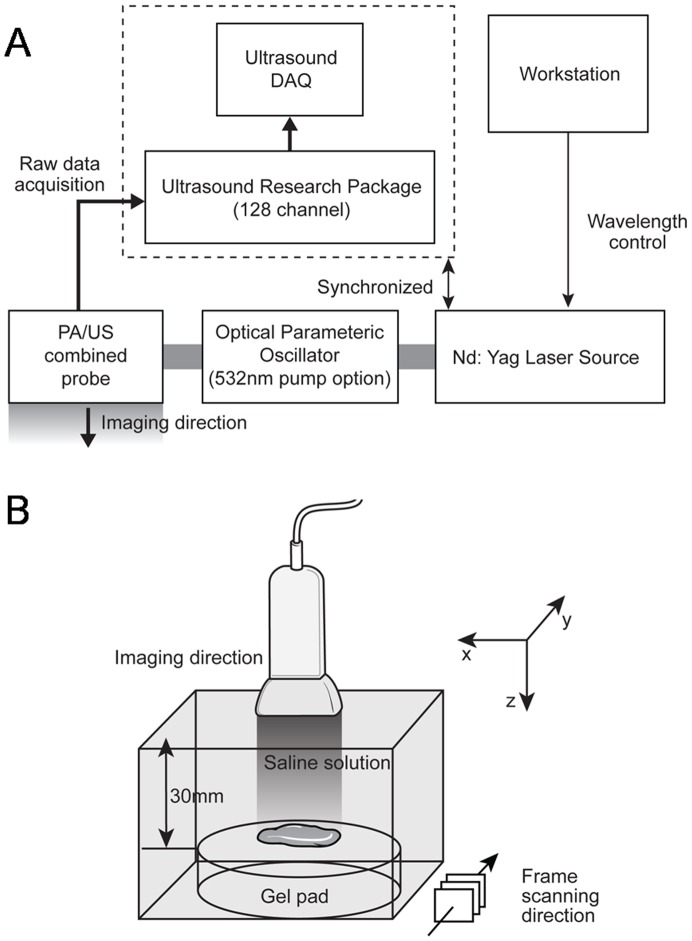
Diagram demonstrates experimental arrangement (A) to acquire photoacoustic signal images from 8-gauge core-biopsied breast specimens (B). To obtain 3-dimensional photoacoustic data, the probe was mechanically moved along the frame scanning direction (B).

The experimental arrangement of the photoacoustic (PA) and US imaging data acquisition system was assembled similar to our previous experiments (Figure 1) [19]. The specimens were set on a scatter-free gel pad (Parker Lab, Inc., Farfield, NJ, USA), which was immersed in a 0.9% saline-filled container. The temperature of the container was stably maintained at 24°C. To acquire PA signals from the specimens, radiofrequency (RF) data were captured with a commercial US scanner equipped with a SonixTouch research package (Ultrasonix Corp., Vancouver, BC, Canada) and a 7-MHz linear array (L14-5/38) connected to a SonixDAQ parallel system. The Q-switch trigger of a Nd:YAG laser excitation system (Surelite III-10 and Surelite OPO Plus, Continuum Inc., Santa Clara, CA, USA) was sent to a SonixTouch research package at 10 Hz of pulse repetition rate. Whenever the US scanner received the scanline-generation trigger, scanline RF data were acquired. The pulse length of the emitted laser was 7 ns and its wavelength was controlled by a software program in a personal computer. The distance between the array transducer and breast specimen was fixed at 30 mm that was longer than the transducer's elevational focal depth of 16 mm. Note that laser delivery was conducted by a custom bifurcated optic fiber bundle and their optical fluency was focused at 30 mm depth from the array transducer (Fiberoptic Systems, Inc., Simi Valley, CA, USA). The imaging reconstruction was conducted with an optimal sound speed of 1500 m/s [20]. Before performing experiments to acquire PA signals, specimens were steeped in saline solution for about 6 hours in order to exclude potentially-remaining blood. This procedure was required when only investigating PA signals generated from microcalcifications without any interference from hemoglobin in blood.

The locations of the breast microcalcifications that appeared in PA images were verified by comparing them with those in specimen mammography. For this, 3-dimensional PA images were acquired through mechanical scanning along the elevation direction of an US array transducer at 0.3 mm increments. For 3-dimensional data, the XYZ-axis mechanical scanner and its driver (SGSP26-100 and SHOT-204MS, Sigma Koki, Co., Ltd., Tokyo, Japan) were implemented with LabVIEW software (National Instruments, Corp., Austin, TX, USA). With the acquired 3-dimensional RF data from the experimental setup, projected PA images in top-down and lateral directions were reconstructed through programmed software of MATLAB (Mathworks, Inc., Natick, MA, USA).

A previous study had demonstrated that microcalcifications in the breast tissue highly absorb laser pulses having a 700 nm wavelength and that the laser energy absorbance consistently decreased from 700 nm to the near infrared range [18]. Based on this result, PA signals were generated by transmitting laser pulses with two different wavelength sections as follows: 700 nm and 800 nm. The researchers performing all experimental procedures were blinded to the findings of specimen mammography.

### Data Analyses

After the experiments were done, 2 radiologists blindly reviewed 3-dimensional PAI data of each core in order to identify microcalcifications within the core and to verify their locations. The presence and location of microcalcifications in PA images of each core were determined when the two radiologists reached an agreement. They found foci that showed PA signals with maximal amplitude on 700 nm and decreased or disappeared PA signals on 800 nm. Each core was then placed into one of two groups after completion of the blind review: those with assumed microcalcifications detected on PA images or those with no microcalcifications identified on PA images. The presence and location of microcalcifications appearing in PA images were demonstrated by comparing them with those in specimen mammography. To verify this visual assessment, we measured quantitative PA responses from selected regions of interest (ROIs) which were drawn manually in the core and calculated the ratio of the amplitude occurring at the 700 nm wavelength section to the amplitude occurring at the 800 nm wavelength section of each focus. The ratio was called a PAI ratio in this paper.

Continuous data including size of calcifications are presented as medians (with ranges). Comparisons between the control group and microcalcification group were performed using the Mann-Whitney U test for continuous variables. In the microcalcification group, multiple regression analysis estimated the relationship between the PAI ratio and other variables. Analysis was performed using SPSS statistical software (SPSS Inc., Chicago, IL, ver 20.0), and statistical significance was accepted as p-value <0.05.

## Results

### Baseline Characteristics

The average age of the 16 patients in this study was 43.81 years (24–74 years). The 11 patients included in the microcalcification group were diagnosed as follows: ductal carcinoma in situ (DCIS) (n = 5), fibrocystic change (n = 4), sclerosing adnenosis (n = 1), and columnar cell change (n = 1).

Each focus of the cores assumed to be found with microcalcifications detected on PA images was matched against existing microcalcifications confirmed by specimen mammography. Table 1 summarizes our 21 cases and shows whether microcalcifications were identified or not on PA images and specimen mammography. The number of calcifications-foci of each core in the microcalcification group ranged from 1 to 45 (mean number, 21.73). The median size of calcifications within the cores is also summarized in Tab. 1 and ranges from 130 to 500 µm.

**Table 1:** Baseline characteristics of 21 cases of the control and microcalcification groups.

Case	age	Pathologic diagnosis	Assum-ed calcific-ation on PAI*	Confirmed calcification on specimen mammography		
				Existence	Number	Size*
1	25	Fibroadenoma	No	No	-	-
2	43	Ductal carcinoma in situ	Yes	Yes	10	500 (210–830)
3	25	Fibroadenoma	No	No	-	-
4	24	Fibroadenoma	No	No	-	-
5	39	Fibroadenoma	No	No	-	-
6	31	Fibrocystic change	Yes	Yes	45	310 (190–940)
7	41	Fibroadenoma	No	No	-	-
8	43	Fibrocystic change	Yes	Yes	17	130 (90–220)
9	49	Ductal carcinoma in situ	Yes	Yes	16	160 (70–260)
10	41	Fibroadenoma	Yes	No	-	-
11	42	Fibrocystic change	Yes	Yes	41	325 (160–390)
12	47	Ductal carcinoma in situ	Yes	Yes	27	220 (110–250)
13	24	Fibroadenoma	No	No	-	-
14	48	Ductal carcinoma in situ	Yes	Yes	14	280 (140–430)
15	24	Fibroadenoma	Yes	No	-	-
16	29	Fibroadenoma	No	No	-	-
17	41	Fibroadenoma	No	No	-	-
18	46	Columnar cell change	Yes	Yes	1	230
19	59	Sclerosing adenosis	Yes	Yes	22	220 (80–760)
20	39	Fibrocystic change	No	Yes	29	265 (120–390)
21	74	Ductal carcinoma in situ	Yes	Yes	17	290 (110–1000)

*Median value (range) of the diameter in calcifications (µm).

Abbreviations: PAI, photoacoustic Imaging.

### Detection of Microcalcifications on Photoacoustic Images

**Figure 2:**
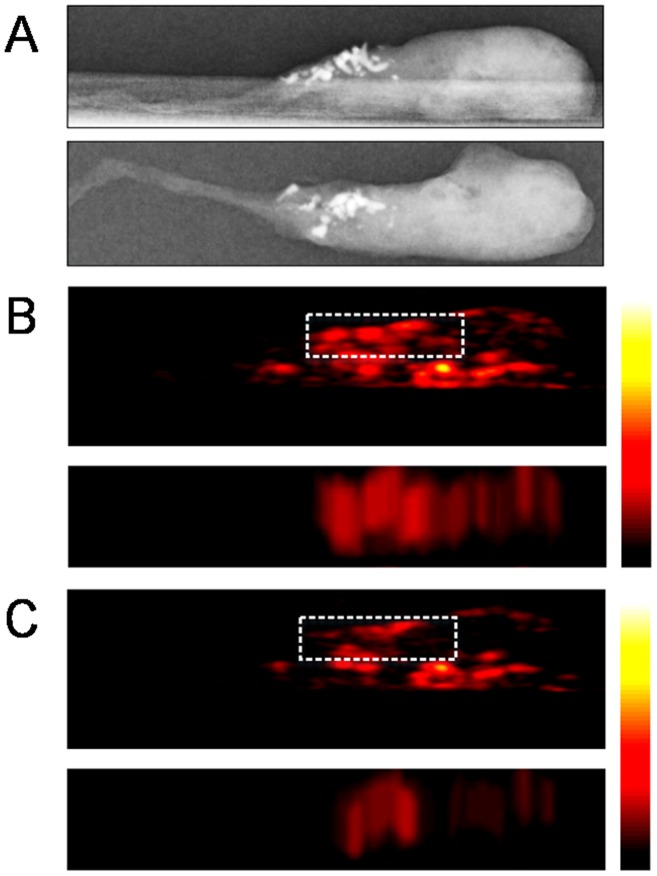
Representative case from ex vivo experiments with core specimens of breast tissue included in the microcalcification group (case #2). Specimen mammography (A) and photoacoustic images obtained from both 700 nm (B) and 800 nm (C) wavelengths were reconstructed with comparable configurations in both directions (top-down and lateral views). The locations of photoacoustic signals were well matched with microcalcifications observed in specimen mammography. The photoacoustic image at 800 nm (C) showed decreasing photoacoustic signal intensities in the region confirmed as microcalcifications through specimen mammography compared with the photoacoustic image at 700 nm (B). The mean value of PAI ratio^†^ from region of interest was 2.80. Abbreviations: PAI ratio, calculated ratio of the amplitude occurring at the 700 nm wavelength section to that occurring at the 800 nm wavelength section.

**Figure 3:**
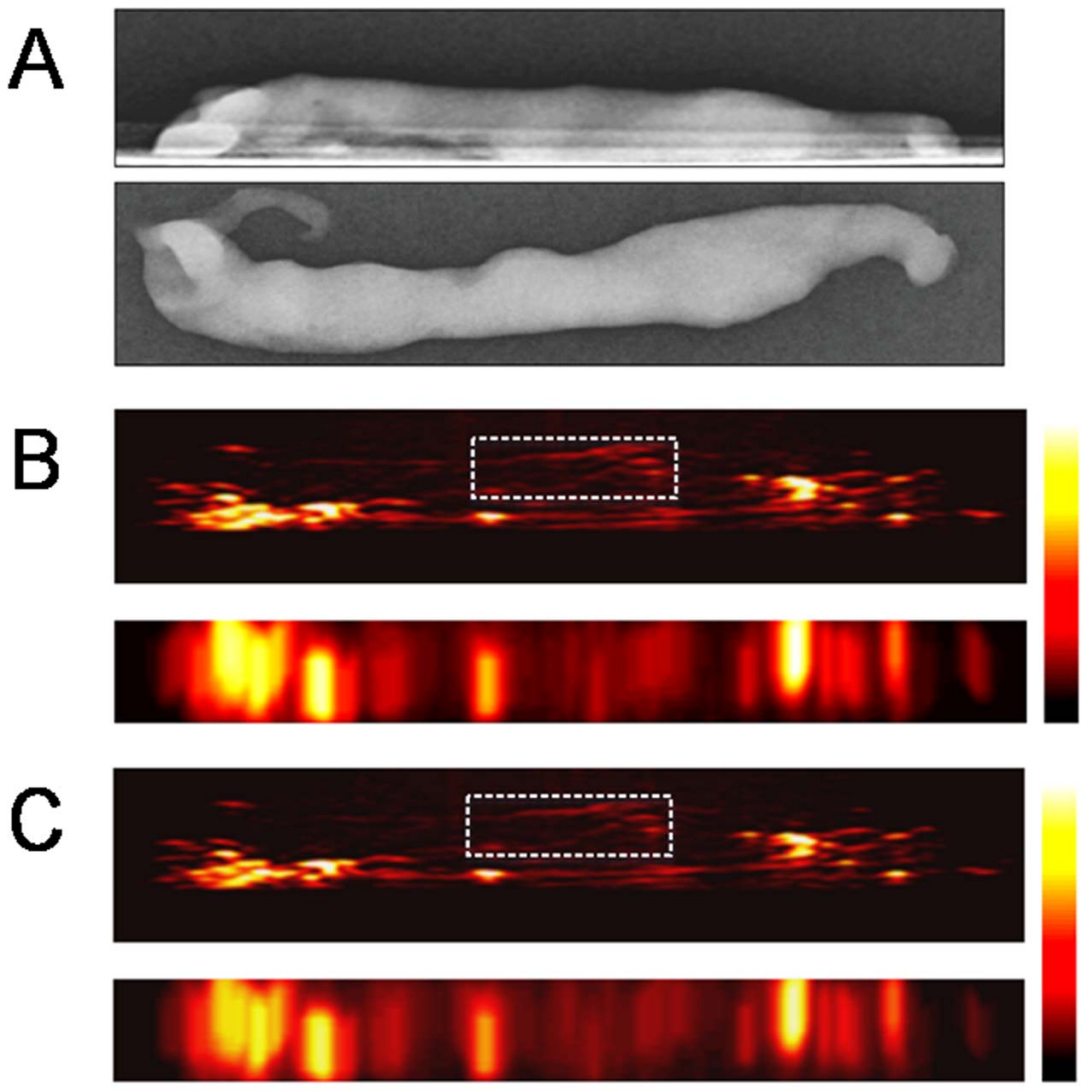
Representative case from ex vivo experiments with core specimens of breast tissue included in the control group (case #5). Specimen mammography (A) and photoacoustic images (B, C) were reconstructed with comparable configurations in both directions (top-down and lateral views). The photoacoustic image at 800 nm (C) shows constant photoacoustic signal intensities from an unidentified target compared with the photoacoustic image at 700 nm (B). The signal foci in the region of interest are assumed to be in the core. All these foci show constant photoacoustic intensities. The mean value of PAI ratio^†^ from region of interest within this core was 1.21. The other bright signal foci around the core may be photoacoustic signals from the interfaces between the surfaces of specimen and the gel pad, which show grossly constant photoacoustic signal intensities. Abbreviations: PAI ratio, calculated ratio of the amplitude occurring at the 700 nm wavelength section to that occurring at the 800 nm wavelength section.

**Figure 4:**
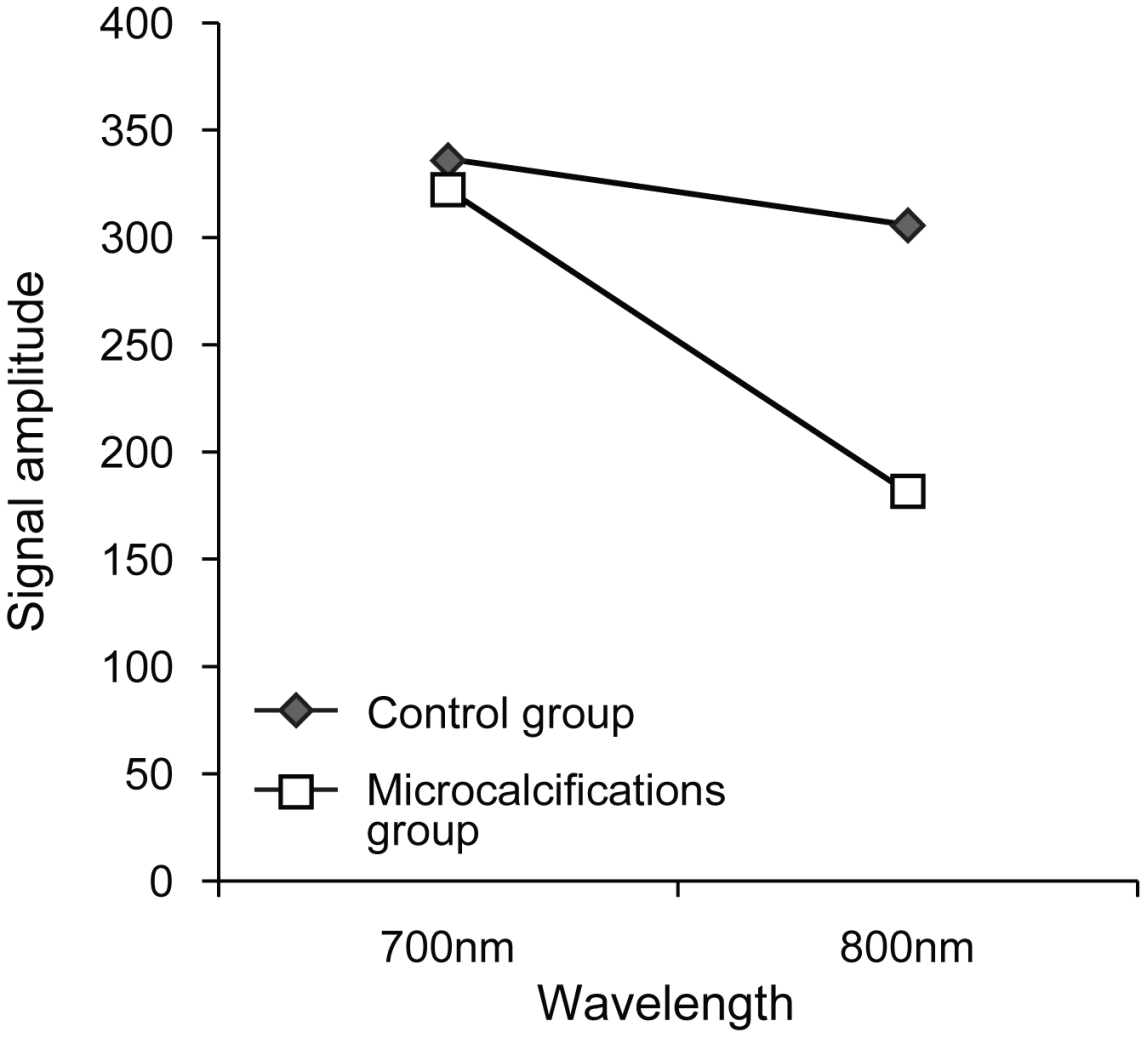
Change in mean values of the maximum photoacoustic signals at the 700 nm and 800 nm wavelength sections in the control and microcalcification group.

Based on the change of PA signal amplitude between 700 nm and 800 nm, 10 out of 11 specimens containing microcalcifications were chosen correctly by the two radiologists identifying the right location of the microcalcifications and 8 out of 10 specimens without calcifications were also identified correctly by the radiologists (Table 2); the sensitivity, specificity, accuracy, positive predictive and negative predictive values of our blinded review were 90.91%, 80.0%, 85.71%, 83.33% and 88.89%, respectively. With regard to the quantitative analysis of PA change, the cores in the microcalcification group showed decreasing PA intensities in the ROI which was proven to be the region containing microcalcifications by specimen mammography (compare Figure 2B and Figure 2C). On the other hand, the cores belonging to the control group showed constant PA intensities from unidentified foci (compare Figure 3B and Figure 3C). Their changes are summarized in Figure 4.

**Table 2:** Results of blind review to assume calcification on PAI.

Presence of Calcification		Calcification on Specimen mammography		Total
		Yes	No	
Calcification on PAI^1^	Yes	10	2	12
	No	1	8	9
Total		11	10	21

Abbreviations: PAI, photoacoustic Imaging.

### Comparison of Quantitative Photoacoustic Responses

**Figure 5:**
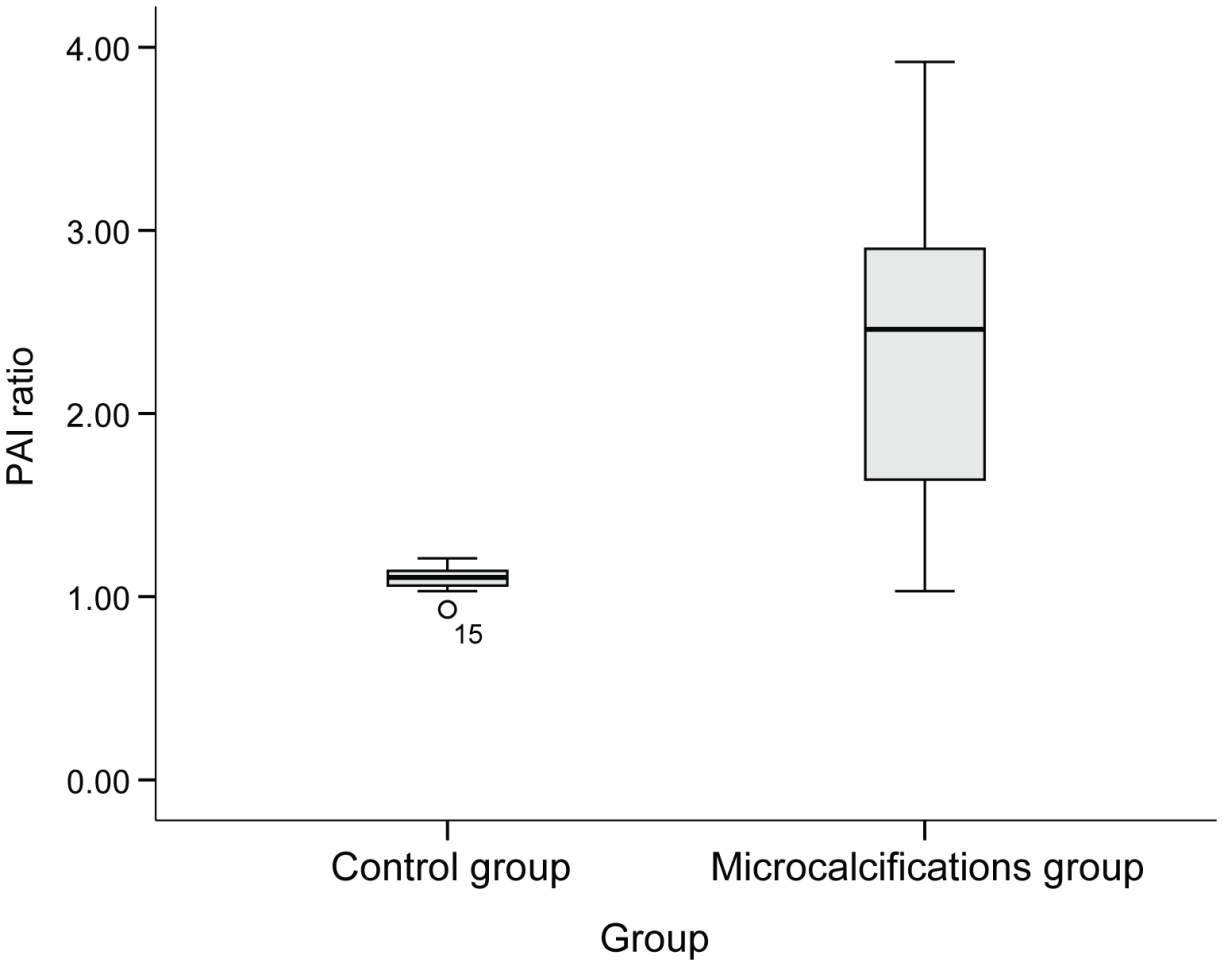
The PAI ratio in the microcalcification group was significantly higher than that in the control group. Abbreviations: PAI ratio, calculated ratio of the amplitude occurring at the 700 nm wavelength section to that occurring at the 800 nm wavelength section.

To verify these changes in PA amplitude, we calculated the PAI ratios of specimens included in the microcalcification group and those of specimens included in the control group. All mean values of PAI ratios from regions of interest within each core are summarized in Table 3. The PAI ratio in the microcalcification group was significantly higher than the PAI ratio of cores included in the control group (median PAI ratio [range], 2.46 [1.03–3.92] versus 1.10 [0.93–1.21], *P* = 0.006), respectively (Figure 5).

**Table 3:** PAI ratios from regions of interest within each core.

Control group		Microcalcification group		*P* value
Case number	PAI ratio	Case number	PAI ratio	
1	1.18	2	2.80	
3	1.14	6	2.63	
4	1.06	8	3.19	
5	1.21	9	1.68	
7	1.08	11	3.0	
10	1.12	12	1.79	
13	1.11	14	2.46	
15	0.93	18	1.03	
16	1.10	19	3.92	
17	1.03	20	1.06	
		21	1.60	
Median value (range)	1.10 (0.93–1.21)	Median value (range)	2.46 (1.03–3.92)	0.006

Abbreviations: PAI ratio, calculated ratio of the amplitude occurring at the 700 nm wavelength section to that occurring at the 800 nm wavelength section.

To find factors that contribute to PAI ratios, we performed an additional subgroup analysis in the microcalcification group. We used a multivariable regression model with the dependent variable of the PAI ratio and the independent predictors of other variables (malignancy, number and size of calcifications-foci). On multiple regression analysis, neither malignant diagnosis nor the number or size of calcification-foci was proven to contribute to PAI ratios (Table 4).

**Table 4:** Multiple regression analysis in the microcalcification group (n = 11) to find factors that contribute to PAI ratios.

	Beta (standard error)	P value
Variables		
Malignant diagnosis*	−0.346 (0.707)	0.639
Number of calcification-foci in the core†	0.001 (0.004)	0.717
Size of calcification-foci in the core†	0.013 (0.028)	0.653

*Malignant result according to the pathology report.

† Median value of calcification-foci in the core.

Abbreviations: PAI ratio, calculated ratio of the amplitude occurring at the 700 nm wavelength section to that occurring at the 800 nm wavelength section.

## Discussion

Visualization of microcalcifications by US examination has been improved due to the development of high resolution US [4], [7], [21]–[23]. However, the current US technology is still considered to be an unreliable method for the detection or evaluation of microcalcifications compared to mammography in clinical use [7], [10], [22]–[27]. A new commercial image processing technique to show more microcalcifications than gray scale US was proposed before, but it was merely an integrated software program that used a filter technique in order to detect only isolated points using higher brightness compared to the surrounding breast tissue [28]. Performers could become confused because of clustered microcalcifications or hyperechoic background including glandular and fibrous tissue [28]. Thus, an imaging technique intrinsically based on microcalcifications is indeed required.

We hypothesized that microcalcifications would have distinguishable absorbance-characteristics from surrounding breast tissue through PA technology and we proved this hypothesis. But, unfortunately, we used biopsy-specimens which were soaked in saline solution causing elimination of blood. Hemoglobin inside breast tissue also has been imaged by PA technology; there may be concern over intervening of hemoglobin when acquiring PA images of microcalcifiations since it is known that oxy-hemoglobin shows increase in absorbance as the wavelength increases from 700 nm to 800 nm and deoxy-hemoglobin demonstrates peak amplitude around 756 nm wavelength. Despite this limitation which is inevitable in the ex vivo study, clinical use of PAI has a promising future since breast microcalcifications have distinguishing absorption spectra in response to different laser emissions [18], [19], [29]–[32]. Further to spectral distinction, we may overcome the limitation applying the principle of Doppler effect or time-of-flight effect in the future in vivo study. Breast microcalcifications is a static image, while hemoglobin makes a kinetic image causing inflow/outflow process. As a result, we expect the problem regarding the flow of blood to be resolved if kinetic images of vascular flow is technically eliminated from the surrounding static images. Therefore, the characteristic absorption spectrum of microcalcifications in response to a specific wavelength of laser pulse and their stationary temper allows PAI to depict distinguishable microcalcifications compared to surrounding in vivo breast.

Previously, 3-dimantional PA images of a couple of benign microcalcification cases were shown to detect breast microcalcifications which were well correlated with detection results of specimen mammography. Further to previous case reports, we proposes the feasibility of PAI with larger sample size including malignant-confirmed microcalcification cases and provides satistical analysis about PA signal intensities at the same time. Curiously enough, the cores of both the microcalcifications and control group presented unidentified PA signal foci because all molecules, including not only microcalcifications but also surrounding tissues in the core, generated PA signals in response to the emitted laser. In addition, unspecified PA signal foci may also originate from the interfaces between the surfaces of the specimen and the saline containing the specimen or the gel pad supporting the specimen. But, these unspecified foci disclosed constant PA signal intensities regardless of wavelength change whereas PA intensities obtained from ROIs of microcalcifications showed characteristic change of PA signal amplitude (median PAI ratio, 1.11 in the control group versus 2.46 in the microcalcification group, *P* = 0.006). As a result, a practical measure to ensure that clear PA images are obtained from just microcalcifications is urgently needed when PAI is applied to human breasts in vivo. Subtraction images can be reconstructed with the difference of PA intensities in microcalcifications. This subtraction which reflects PAI ratio and allows enhancing contrast resolution would be one of the solutions for practical use of PAI.

In this study, with the use of PAI, a sensitivity of 90.91% and a specificity of 80.0% for detection of microcalcifications within the specimens were achieved although false positive (n = 2) and false negative (n = 1) results were found. This may be because the blinded review to find microcalcifications on PA images did depend on the gross judgment of the two radiologists. The subtraction images which might reflect PAI ratio would make it easier for performers to detect microcalcifications and resolve false positive or false negative results. For clinical application of PAI, technically improved transducer with a program exhibiting subtraction images concurrently should be developed to emphasize and maximize contrast between microcalcifications and surrounding tissues.

There have been several attempts to identify underlying biochemical differences that distinguish benign from malignant microcalcifications, but a definite difference in chemical composition has not been clarified yet [33]–[37]. Two major types of microcalcifications found in breast tissue (calcium oxalate and hydroxyapatite) have been investigated by Raman spectroscopy [11], [38]–[40]. Although only benign calcification cases were included, the possibility of different PAI ratios between malignant and benign microcalcifications was mentioned through phantom experiments of calcium oxalate and hydroxyapatite [19]. Instead of analyzing the chemical composition inside the cores, we tried to find factors contributing to different PA signal amplitudes of calcifications, especially including malignant diagnosis as one of the independent predictors. No significant factors were found among variables such as malignant diagnosis as well as the number and size of calcification-foci in the subgroup analysis (n = 11). In order to clarify the difference of PAI ratio according to the intrinsic character of calcifications, we will need to perform PAI with increased sample size and should measure the quantitative PA response of calcium oxalate and hydroxyapatite in the future; their absorbance features might differentiate each other, which is consistent with previous reports about calcium oxalate and hydroxyapitite in breast lesions [3], [33], [38], [41].

Our study still has a number of limitations. First of all, our sample size is relatively small as it is one of the preliminary studies about PAI of breast microcalcifications. Therefore, further studies with larger sample sizes will be necessary. Second, since this is a pilot study with biopsy specimens, practical application might still be limited. Further prospective studies should be performed by developing a sophisticated PA transducer and controlling unexpected properties of PA signals that might occur in its in-vivo use.

In conclusion, breast microcalcifications generated distinguishable photoacoustic signals unlike breast tissues without calcifications. So, PAI, a non-ionizing and non-invasive hybrid imaging technique, can propose an alternative in overcoming the limitations of the conventional US imaging.
